# Targeting and Specific Activation of Antigen‐Presenting Cells by Endogenous Antigen‐Loaded Nanoparticles Elicits Tumor‐Specific Immunity

**DOI:** 10.1002/advs.201900069

**Published:** 2019-11-08

**Authors:** Hao‐Cai Chang, Zheng‐Zhi Zou, Qiu‐Hong Wang, Jie Li, Huan Jin, Qian‐Xia Yin, Da Xing

**Affiliations:** ^1^ MOE Key Laboratory of Laser Life Science & Institute of Laser Life Science South China Normal University Guangzhou 510631 China; ^2^ College of Biophotonics South China Normal University Guangzhou 510631 China

**Keywords:** antigen‐presenting cells, cancer immunotherapy, CD8^+^/CD4^+^ T cells, codelivery system, HSP70‐chaperoned polypeptides

## Abstract

Immunotherapy has shown tremendous promise for improving cancer treatment. Unfortunately, antigen‐presenting cells (APCs) in cancer patients cannot effectively recognize and process tumor antigens to activate host immune responses. In this study, an approach is developed to improve cancer immunotherapy that utilizes endogenous antigen‐carrying nanoparticles (EAC‐NPs), which encompasses a set of antigens isolated from solid tumors and adjuvants. The EAC‐NPs specifically target APCs and subsequently result in enhanced T cell responses and improved antitumor efficacy. Mechanistic studies reveal that the EAC‐NPs enhance and prolong the presence of immune compounds in APCs, which ensure persistent antigen loading and stimulation, induce a rapid proliferation of CD4^+^ and CD8^+^ T cells, and significantly increase the ratios of intratumoral CD4^+^ T/T_reg_ and CD8^+^ T/T_reg_. The work using nanotechnology provides a promising strategy in improving antitumor immunity by enhancing the immunogenicity and presentation of tumor self‐antigens for cancer immunotherapy.

## Graphical abstract

Endogenous antigen‐carrying nanoparticles are phagocytosed by antigen‐presenting cells (APCs) triggering antigen and Toll‐like receptor signaling. Then the APCs induce highly efficient differentiation of T cells toward helper T (CD4^+^) cells and cytotoxic T lymphocytes (CD8^+^) to finally generate potent antitumor immune responses. A promising strategy is established for cancer immunotherapy by enhancing the immunogenicity and presentation of tumor self‐antigens.

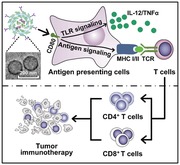

## 1 Introduction

Cancer immunotherapy, a promising and powerful approach, has been introduced into clinical practice.^1^ Specific immune response system can effectively recognize and eliminate tumor cells, and prevent their metastasis and recurrence.^2^ Dendritic cells (DCs) and monocytes^3^/macrophages, as antigen‐presenting cells (APCs), play essential roles in specific immune response for cancer immunotherapy. DCs and monocytes/macrophages take up and process antigens and then present them to T cells via major histocompatibility complex class I and II (MHC I and II) molecules.^4^ Activated and mature DCs or macrophages can induce the responses of cytotoxic T lymphocytes (CTLs), which are crucial for eradicating tumor cells.^5^ The amplification of CTL responses depends on antigen loading and activation of APCs.^6^ However, many therapeutic cancer strategies may not meet these requirements,^7^ and any missteps in antigen processing and presentation will diminish efficacy or may induce antigen‐specific tolerance.^8^

APC activation in immunotherapy is challenging for several reasons. One reason is the inadequate presentation of tumor antigens, where tumor cells themselves are nonimmunogenic and express little or no MHC I or II.^9^ Another problem is lack of pattern‐recognition receptor signaling including the Toll‐like receptor (TLR) signaling, which controls the expression of many innate response genes and can directly induce the activation of APCs.^10^ A third challenge is that antigens and adjuvants (producing TLR signaling) may be inefficiently processed by APCs,^11^, ^12^ whose ability to elicit an immune response is thereby limited. A fourth challenge is that there may be a disconnect between the influx of antigens and adjuvants into the same APCs at the same time.^11^ A fifth problem is that the activity of immune cells, particularly APCs, may be suppressed by various factors in the tumor microenvironment.^13^ Most importantly, patient‐specific neoantigens lacking in immunotherapy are still a major challenge.^12^

Codelivering antigens and adjuvants to APCs may induce stronger immune responses. However, the tumor antigen is usually inadequate, and the adjuvant molecules are usually composed of microbial products that need to be injected into the body. This highlights the need for 1) effective acquisition of tumor antigens; 2) appropriate nanocarriers that can deliver both antigens and adjuvants protect them from degradation in vivo and ensure synchronized delivery to APCs; and 3) controlled release of antigens and adjuvants in APCs to ensure maximization of their activity.

Activation of adaptive immune responses by presenting endogenous antigens is an effective way to kill tumors and can simultaneously generate memory immune responses. However, for many tumors, most endogenous tumor antigens are unknown. Studies have reported that heat shock protein 70 (HSP70) acts as a chaperone for several polypeptides that can generate tumor antigens, which can be represented by MHC molecules^14^ and elicit CD8^+^ T cell responses.^15^ The immunogenic nature of the HSP70‐chaperoned polypeptides (HCP) allows for their novel use in personalized immunotherapy of cancer. Because an effective adaptive immune response is needed for TLR signaling,^16^ oligodeoxynucleotides (ODNs) containing demethylated CpG motifs (CpG ODNs), as an intracellular ligand of TLR9, have been utilized to induce the maturation of DCs^17^ and monocytes,^18^ whose maturation is to extravasate into tissue and to differentiate into macrophages and DCs.^19^ Additionally, CpG ODNs act as adjuvants to boost protein‐based immunogens for antigen‐specific immune responses^20^ and overcome antitumor immune tolerance.^21^ The combination of HSP70‐chaperoned polypeptides and CpG ODNs may be an optimal choice to treat tumors.

Various protein nanocages, including encapsulin,^22^ ferritins,^23^ virus‐like particles,^24^ and lumazine synthase,^25^ have been extensively studied as nanoscale vehicles for targeted drug delivery. In particular, poly (ε‐caprolactone)‐block‐poly (ethylene glycol) (PCL‐b‐PEG) is a biocompatible and biodegradable polymer that is widely used in drug delivery.^26^ A hydrazone (Hyd) bond introduced by modification of the amphipathic ring of PCL‐b‐PEG can be hydrolyzed in the mildly acidic pH conditions (pH 5–6) of the tumor microenvironment or endosomes and can cause nanocages to collapse.^27^

**Figure 1:**
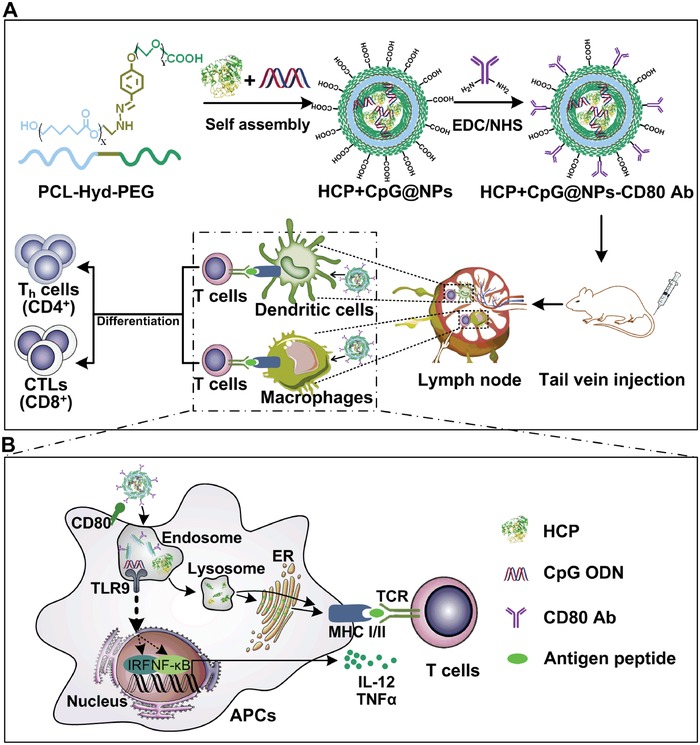
Schematic illustration of the EAC‐NP formation and the mechanisms of EAC‐NP‐induced cancer immunotherapy. A) Schematic illustration of the preparation of HCP+CpG@NPs‐CD80 Ab vesicles and induction of T‐cell immune responses. B) Partial magnification of (A). Once phagocytosis occurs, APCs are activated through two signaling pathways: antigen signaling and TLR signaling. After activation, APCs deliver antigen signaling to T lymphocytes, which differentiate into helper T (Th) cells and CTLs, even produce memory T cells.

Herein, we constructed novel pH‐responsive biodegradable PCL‐Hyd‐PEG vesicles encapsulating tumor endogenous antigens HCP and adjuvants CpG ODN, which could efficiently activate APCs, and then trigger CTL and long‐term memory immune responses. Vesicle shells were modified with an antibody against CD80 (CD80 Ab) (**Figure** **1**), which is expressed on APCs, including DCs^28^ and monocytes.^29^ The therapeutic efficacy of endogenous antigen‐carrying nanoparticles (EAC‐NPs) was evaluated in subcutaneous mastadenoma model and pulmonary metastatic melanoma model. Importantly, the mechanisms governing the antitumor response induced by the nanoparticles were also explored.

## 2 Results and Discussion

### 2.1 Tumor‐Specific Preparation and Characterization of HCP

**Figure 2:**
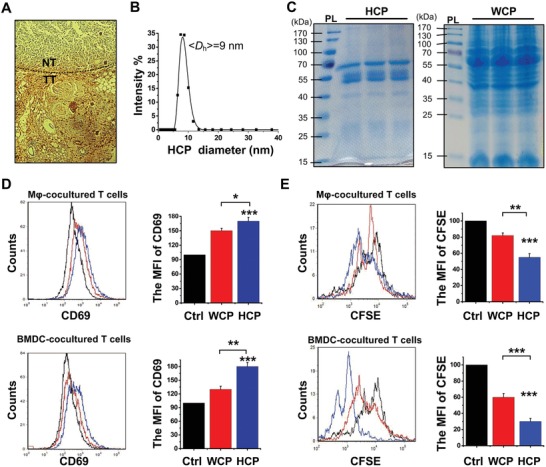
The characterization of HCP. A) The expression of HSP70 on 4T1 tumor tissue (TT) and normal tissue (NT) was analyzed by immunohistochemistry. B) Size distribution of the isolated HCP. C) The isolation of HCP (left) and whole‐cell protein (WCP) (right) were separated by SDS‐PAGE gels and then the gels were stained using Coomassie blue. The left band of each panel is the protein ladder (PL). D) The expression of CD69 on activated T lymphocytes was analyzed by flow cytometry after coculturing with different treated Mφ (upper panel) and BMDCs (bottom panel) for 18 h. E) The proliferation of T lymphocytes with CFSE staining was analyzed by flow cytometry after coculturing with different treated Mφ (upper panel) and BMDCs (bottom panel) for 72 h. MFI: Mean fluorescence intensity. *P < 0.05, **P < 0.01, and ***P < 0.001.

The expression of HSP70, a chaperone protein, in tumor tissue and normal tissue was confirmed by immunohistochemistry using an anti‐HSP70 antibody. The results showed that HSP70 had a relatively high expression in tumor tissue (**Figure** **2**A), implying presence of an abundance of tumor antigens from the chaperoned polypeptides. HCP was then generated from homogenized lysates of tumor tissues. ≈40 µg of HCP was obtained per gram of tumor tissue. To characterize HCP, size distribution was measured by dynamic light scattering (DLS), and Coomassie blue staining of gels was used to confirm the molecular weight range of HCP. The results showed that the size distribution of HCP was ≈9 nm (Figure 2B), and the molecular weight was 50–70 kDa (Figure 2C). Additionally, HCP‐treated macrophages (Mφ) and bone marrow‐derived dendritic cells (BMDCs) significantly upregulated the CD69 expression (Figure 2D), an early activation marker of T lymphocytes, and increased the proliferation of T lymphocytes (Figure 2E). These results suggest that the isolated HCP may be a highly immunogenic tumor antigen.

### 2.2 Construction and Characterization of EAC‐NPs

**Figure 3:**
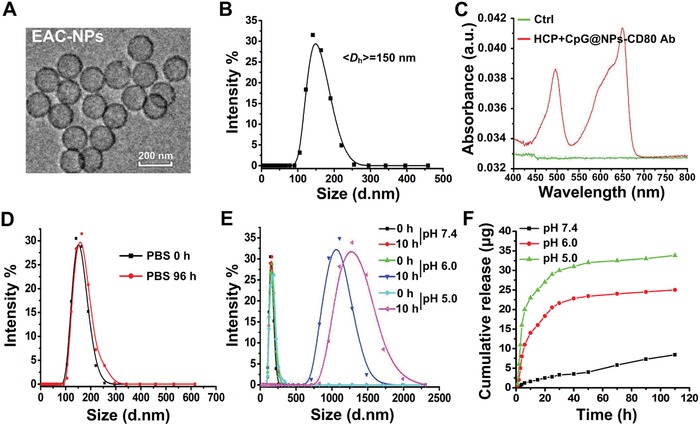
Characterization of EAC‐NPs. A) Transmission electron microscopy images of EAC‐NPs. B) Size distributions of EAC‐NPs at 0.4 mg mL^−1^ determined by DLS at 25 °C. C) The absorbance spectrum of the EAC‐NPs with CpG‐FITC (488 nm) and CD80 Ab‐APC (650 nm). D) Size distribution of EAC‐NPs in PBS (0.01 m, pH 7.4) at different time points. E) Size distribution in 0.01 m PBS at pH 5.0, 6.0, and 7.4 after 10 h. F) The cumulative release of HCP from 2 mg EAC‐NPs at different time points in the supernatant was measured using the Bradford assay in 0.01 m PBS at pH 5.0, 6.0, and 7.4.

The synthesis of EAC‐NPs (HCP+CpG@PCL‐Hyd‐PEG‐CD80 Ab NPs) is described in Figure 1. Transmission electron microscopy images of EAC‐NPs showed vesicular morphology (**Figure** **3**A). In addition, DLS experiments were performed to analyze vesicle size distribution of self‐assemblies as shown in Figure 3B. At pH 7.4, the mean hydrodynamic diameter of the vesicles with CD80 Ab was 150 nm at 0.4 mg mL^−1^ of the PCL‐Hyd‐PEG copolymer. Absorption spectroscopy of the vesicles (Figure 3C) revealed absorption peaks at 488 and 650 nm, suggesting a successful modification of the vesicles with CpG (FITC fluorophore) and CD80 Ab (APC fluorophore). The surface potential of the vesicles decreased from −10 ± 2.5 to −15 ± 3.3 mV when the vesicles were modified with CD80 Ab (Table S1, Supporting Information). This prevented the vesicles from being taken up in the liver (Figure S1, Supporting Information) as negative zeta potential particles have higher stability in circulation in comparison to positive potential particles.^30^ In addition, the encapsulation efficiency of HCP and CpG was 90.3 ± 4.2% and 91.5 ± 3.0%, respectively.

To evaluate the physiological stability of EAC‐NPs, the NPs in PBS (0.01 m, pH 7.4), Dulbecco's modified Eagle's medium (DMEM), fetal bovine serum (FBS), and DMEM with FBS (10%) were monitored by measuring vesicle size and zeta potential in vitro for more than 90 h. As shown in Figure 3D and Figure S2 (Supporting Information), when the EAC‐NPs were placed in different solutions, there were no obvious size and zeta potential changes. The biological compatibility and stability of EAC‐NPs in solution suggest that the vesicles are a promising fit for the intended in vivo application. To determine how pH changes affected the Hyd bond and subsequent antigen and adjuvant release, vesicle sizes were measured by DLS at different pH values (0.01 m PBS, pH 5.0, 6.0, and 7.4). The results showed that the vesicles rapidly and remarkably swelled (Figure 3E), and then gradually collapsed (Figure S3, Supporting Information). HCP concentration in the supernatants at the different pHs was measured at different time points using Bradford assay. As depicted in Figure 3F, the release of HCP from the degrading vesicles was greater and faster at pH 5.0 and pH 6.0 than at pH 7.4, suggesting that pH could effectively control the release of protein from the vesicles.

### 2.3 Delivery of EAC‐NPs into APCs

**Figure 4:**
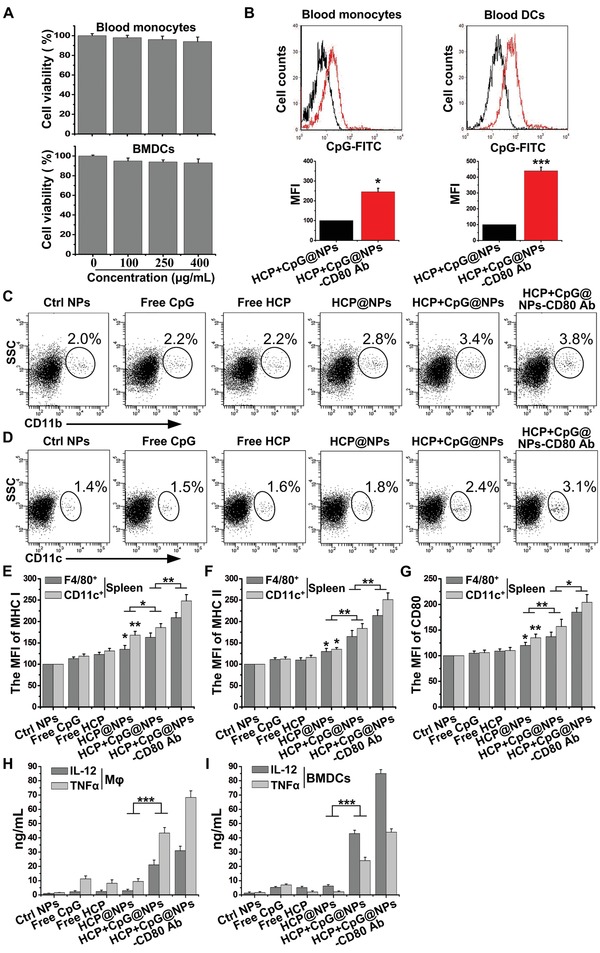
The effect of EAC‐NPs on monocytes/macrophages and DCs. A) Cell viability of blood monocytes and BMDCs incubated with different concentrations of HCP+CpG@NPs‐CD80 Ab for 10 h and with fresh serum‐supplemented media for another 48 h before CCK‐8 assay. B) The cellular uptake of nanoparticles (CpG‐FITC) in blood CD11b^+^ monocytes and CD11c^+^ DCs was assessed by flow cytometry at 12 h after the intravenous injection of HCP+CpG@NPs or HCP+CpG@NPs‐CD80 Ab. The abundance of C) CD11b^+^ monocytes and D) CD11c^+^ DCs from peripheral blood was analyzed by flow cytometry at 24 h after the intravenous injection of different treatments. The MFI of E) MHC I, F) MHC II, and G) CD80 expression on F4/80^+^ macrophages and CD11c^+^ DCs from spleen was analyzed by flow cytometry at 36 h after the intravenous injection of different treatments. H) Mφ and I) BMDCs were incubated EAC‐NPs (400 µg mL^−1^) for 2 h and washed, then the release of IL‐12 and TNF‐α in the supernatant of drug‐free culture medium for 22 h was measured by ELISA. MFI: Mean fluorescence intensity. *P < 0.05, **P < 0.01, and ***P < 0.001.

To quantitatively evaluate the potential toxicity of combined intravenous administration of antigens and adjuvants, cell viability was assessed for blood monocytes and BMDCs with different vesicle concentrations. The results in **Figure** **4**A showed that the cells incubated with EAC‐NPs (up to 400 µg mL^−1^) maintained viability up to 90%, indicating that the vesicles were low toxic to APCs. Then, the cellular uptake of nanoparticles in blood monocytes and DCs in vivo was assessed by flow cytometry with or without the targeted molecules CD80 Ab. The results showed that the phagocytosis of EAC‐NPs by blood monocytes and DCs was significant increases in the presence of the targeted molecules CD80 Ab (Figure 4B), suggesting that CD80 Ab promoted EAC‐NPs to effectively target blood monocytes and DCs. Then, the abundance of monocytes and DCs from peripheral blood was examined using flow cytometry. The administration of EAC‐NPs for immunotherapy significantly increased the abundance of CD11b^+^ monocytes (Figure 4C) and CD11c^+^ DCs (Figure 4D) from peripheral blood, which implies increased systemic T cell activation in these animals. Importantly, the expression of MHC I, MHC II, and CD80 on splenic F4/80^+^ macrophages and CD11c^+^ DCs, which were contributed by blood monocytes and DCs, was significantly upregulated by functional vesicles in vivo (Figure 4E,F,G). This resulted in a potential increase in the formation of MHC‐peptide complexes, which shaped the T cell repertoire.^31^ CpG has been reported to activate APCs and then induce the secretion of IL‐12 and TNF‐α,^32^ which promote the differentiation of antigen‐specific effector T cells.^33^ Therefore, we detected the secretion of IL‐12 and TNF‐α by macrophages and DCs after treatment with different modified vesicles. CpG‐containing vesicles induced greater secretion of IL‐12 and TNF‐α than vesicles without CpG (Figure 4H,I). More importantly, a combination of HCP, CpG, and CD80 Ab in the vesicles further enhanced the release of IL‐12 and TNF‐α, suggesting that more antigen‐specific effector T cells were produced.

### 2.4 T Cell‐Mediated Immunity Induced by EAC‐NPs

**Figure 5:**
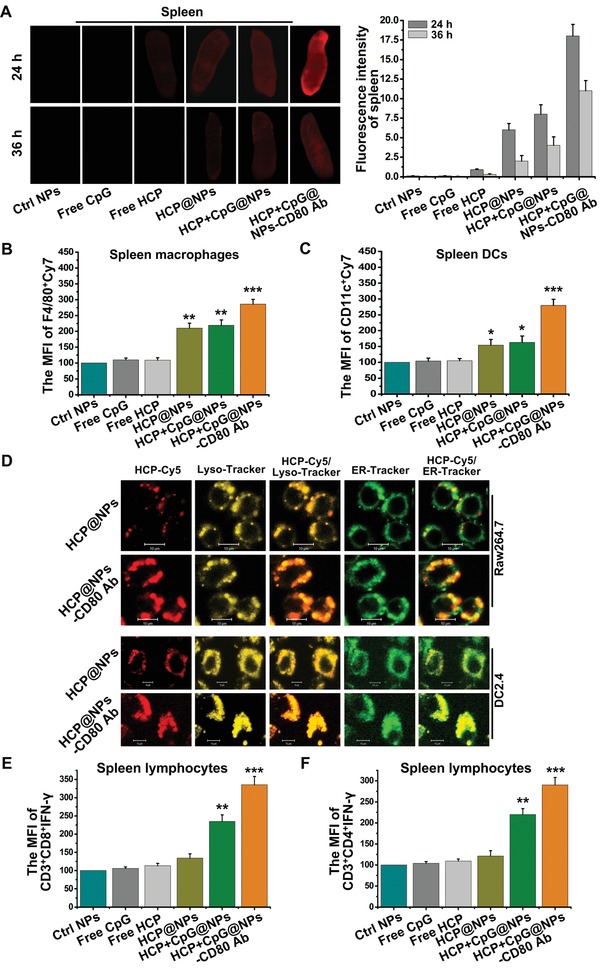
The accumulation of NPs and activation of T lymphocytes in the spleen. A) Ex vivo imaging of spleens from mice 24 and 36 h after intravenous injection of different modified NPs (HCP labeled by Cy7‐NH_3_) was visualized and the fluorescent images of spleens were quantitatively analyzed using an Odyssey infrared imaging system. B) F4/80^+^ macrophages and C) CD11c^+^ DCs with modified NPs (Cy7) in spleens were quantified by flow cytometry at 24 h after different treatments (*n* = 4). D) Confocal images of RAW264.7 and DC2.4 cells at 2 h after treatment with HCP+CpG@NPs or HCP+CpG@NPs‐CD80 Ab. Scale bar: 10 µm. Flow cytometry analysis evaluated the IFN‐γ expression on E) CD3^+^CD8^+^ and F) CD3^+^CD4^+^ T cells in spleens of animals 16 d after tumor‐inoculation under different treatments (*n* = 4). *P < 0.05, **P < 0.01, and ***P < 0.001.

After antigen presentation by macrophages and DCs in lymphoid tissues, the antigen‐experienced T cells are allowed clonal expansion and then acquire helper capabilities (Th cells) or specific cytotoxic functions (CTLs), even produce memory properties (memory T cells). Systemic injection of NPs can enhance the number of splenic macrophages and DCs and initiate T‐cell responses.^34^ To determine whether administration EAC‐NPs loaded with HCP (HCP labeled by Cy7‐NH_3_) would accumulate in spleen and lymph node via phagocytosis by macrophages and DCs, and induce the activation of T cells, the distribution of HCP‐Cy7 and the expression of IFN‐γ on T cells in spleen were assessed in tumor‐bearing animals. We found that 24 h after treatment, there was significant fluorescence intensity in spleens of EAC‐NP‐treated animals in comparison to those of not treated with EAC‐NPs (**Figure** **5**A). These results indicate that there was greater phagocytosis of EAC‐NPs by blood monocytes and DCs, which subsequently migrated to spleen and lymph node (Figure 5A and Figure S4A,B, Supporting Information). After 36 h, the fluorescence signal disappeared from blood (Figure S4C, Supporting Information), but spleen and lymph node still had a strong fluorescence signal, suggesting that the vesicles prolonged the retention of the immune compounds by APCs in lymphoid tissues. We further quantified the amount of Cy7^+^ EAC‐NPs‐loaded F4/80^+^ macrophages and CD11c^+^ DCs in spleen by flow cytometry. The results showed that the phagocytosis of EAC‐NPs by macrophages (Figure 5B) and DCs (Figure 5C) in spleen was significantly increased.

Exogenous antigens are processed by the lysosome (Lyso) and endoplasmic reticulum (ER) and then presented by APCs.^35^ Cy5‐labeled HCP (HCP‐Cy5) was encapsulated in the vesicles, and then the vesicles were incubated with RAW264.7 and DC2.4 in the absence of serum for 2 h. The distribution of HCP‐Cy5 was then observed using confocal laser scanning microscopy after the vesicles were engulfed by the cells. Cellular uptake of HCP‐Cy5 increased and localized more to the Lyso and ER when the vesicles were coated with CD80 Ab (Figure 5D). This suggests that CD80 Ab promoted NPs to effectively target APCs and that immunogenic HCP could be processed into antigenic peptides. Additionally, we found that the expression of IL‐12 and TNF‐α in splenic F4/80^+^ macrophages and CD11c^+^ DCs was significantly induced by CpG‐containing vesicles, compared with the treatment of vesicles without CpG (Figure S5, Supporting Information). Subsequently, spleen lymphocytes were isolated from animals under different treatments, and the expression of IFN‐γ on CD3^+^CD8^+^ (Figure 5E) and CD3^+^CD4^+^ (Figure 5F) T cells from treatment with EAC‐NPs was vastly upregulated. This indicates that HCP was processed into antigenic peptides and these EAC‐NPs further promoted the production of a tumor‐specific immune response.

### 2.5 Multifunctional EAC‐NPs Induce Antigen‐Specific Immunity and Tumor Inhibition in Mice

**Figure 6:**
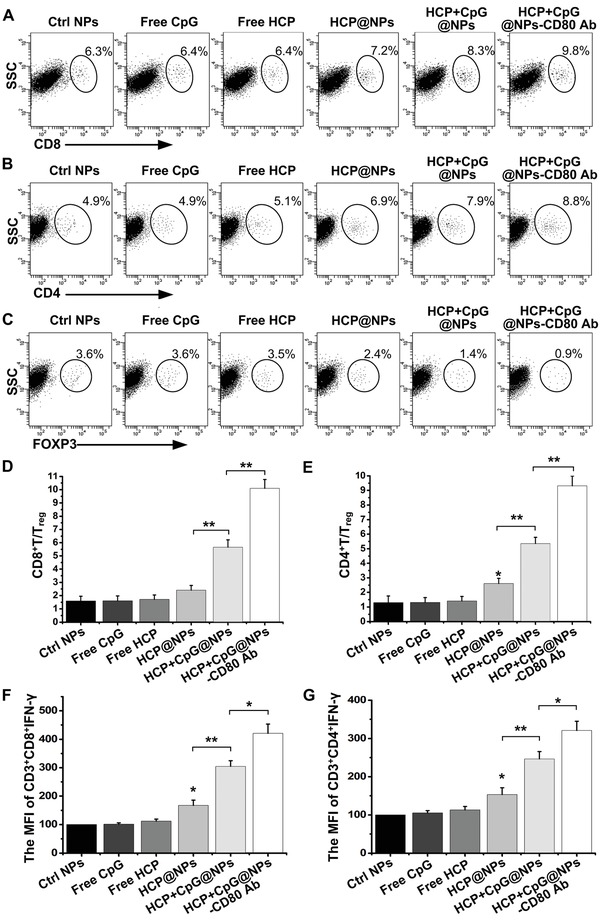
EAC‐NPs enhanced intratumoral immunity. 4T1 tumor tissues were harvested 18 d after the different treatments. Flow cytometry analysis was used to assess the abundance of A) CD8^+^ T cells, B) CD4^+^ T cells, and C) CD4^+^CD25^+^FOXP3^+^ T cells in 4T1 tumors of animals treated with different modified NPs 18 d after tumor inoculation (*n* = 4). The T cells in this assay were defined as CD3^+^. The ratios of intratumoral D) CD8^+^ T/T_reg_ and E) CD4^+^ T/T_reg_ from (A) to (C). The MFI of IFN‐γ expression on the F) CD3^+^CD8^+^ T cells and G) CD3^+^CD4^+^ T cells in EMT6 tumors of animals under different treatments was analyzed by flow cytometry (*n* = 4). MFI: Mean fluorescence intensity. *P < 0.05, **P < 0.01, and ***P < 0.001.

To determine whether activated T lymphocytes in lymphoid tissues would migrate to tumor tissue, the abundance of tumor‐infiltrating lymphocytes was detected in EMT6 and 4T1 tumor models. EMT6 or 4T1 cells were subcutaneously inoculated into mice on day 0, and then EAC‐NPs were injected intravenously on days 4, 6, and 8. We found that in comparison to control group, the animals treated with EAC‐NPs had more CD3^+^CD8^+^ T cells (**Figure** **6**A) and CD3^+^CD4^+^ T cells (Figure 6B) and fewer CD3^+^CD4^+^CD25^+^FOXP3^+^ T_reg_ cells (Figure 6C) 18 d post‐tumor inoculation. The data strongly suggest that the addition of the EAC‐NPs significantly increased the ratios of intratumoral CD8^+^ T/T_reg_ (Figure 6D) and CD4^+^ T/T_reg_ (Figure 6E), thereby increasing the antitumor immunotherapeutic capacity. In addition, we found that tumors isolated from animals in EAC‐NP treatment group on day 18 post‐tumor‐inoculation had the most robust IFN‐γ production by CD8^+^ (Figure 6F) and CD4^+^ (Figure 6G) T cells. Particularly, the marked increase of CD8^+^CD44^+^CD122^+^ (Figure S6A,C, Supporting Information) and CD4^+^CD44^+^CD122^+^ (Figure S6B,D, Supporting Information) memory T cell proportion in EAC‐NP treatment group compared with other treatment groups indicated that an excellent permanent immunity was established against the corresponding tumor. Collectively, our results demonstrated that the formed vesicles increased the anticancer quantity and quality of CD4^+^ and CD8^+^ effector antigen‐specific T cells.

**Figure 7:**
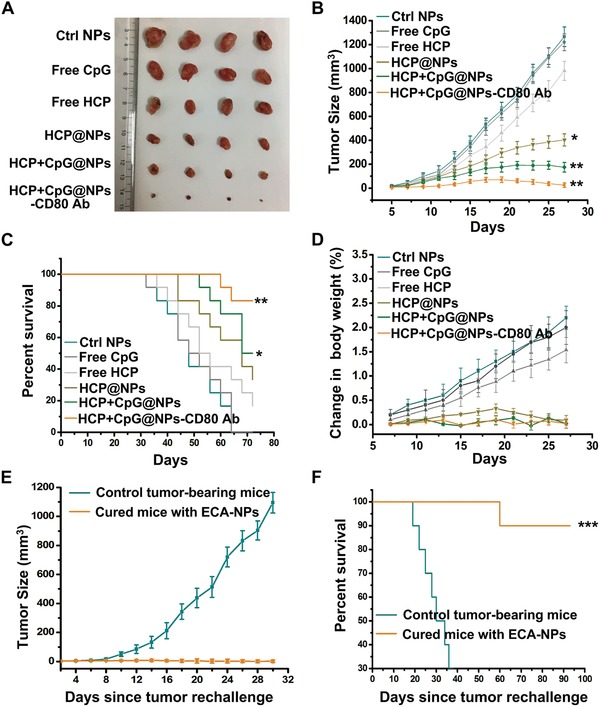
EAC‐NPs caused tumor regression. A) Representative images of 4T1 tumors collected from tumor‐bearing mice following 27 d of treatment as indicated. B) Tumor growth curves of tumor‐bearing mice treated with different modified NPs every two days over 27 d (*n* = 16). The data show mean ± SD. C) Survival curves of tumor‐bearing mice treated with different modified NPs (*n* = 12). D) Analysis of body weight change in tumor‐bearing mice in (B) (*n* = 8). E) Tumor growth curves of individual animals cured 90 d after EMT6 tumor inoculation using the EAC‐NPs and then rechallenged with EMT6 tumor cells (*n* = 14). Mice of the same age were inoculated with tumor cells as controls. The data show mean ± SD. F) Survival curves of tumor‐bearing mice in (E) (*n* = 10). Differences in survival were determined using the log‐rank test. *P < 0.05, **P < 0.01, and ***P < 0.001.

Finally, to confirm the therapeutic efficacy of EAC‐NPs, tumor size and mouse survival were monitored every 2 d. We found that the administration of the formed EAC‐NPs significantly delayed tumor growth (**Figure** **7**A,B) and increased survival time (Figure 7C). Additionally, there was no significant change in the body weight of mice with different treatments (Figure 7D), suggesting that all experimented mice were healthy. To further confirm the permanent immunity elicited by the formed EAC‐NPs, mice cured 90 d after tumor inoculation using EAC‐NPs were rechallenged with tumor cells. Impressively, the cured mice were significantly resistant to tumor rechallenge (Figure 7E), and 90% of the cured mice were still alive on day 80 after tumor rechallenge (Figure 7F). Taken together, the in vivo data suggest that the EAC‐NP treatment resulted in enhancement of tumor‐specific T cells and durable antitumor immunity. To assess toxicity, hematoxylin and eosin (H&E) stained tissue sections from healthy mice that underwent different treatment regimens were evaluated (Figure S7, Supporting Information). Macroscopically, no noticeable lesions were observed in the major organs, suggesting a reasonable safety profile for EAC‐NPs.

### 2.6 EAC‐NPs Inhibit Tumor Metastasis In Vivo

**Figure 8:**
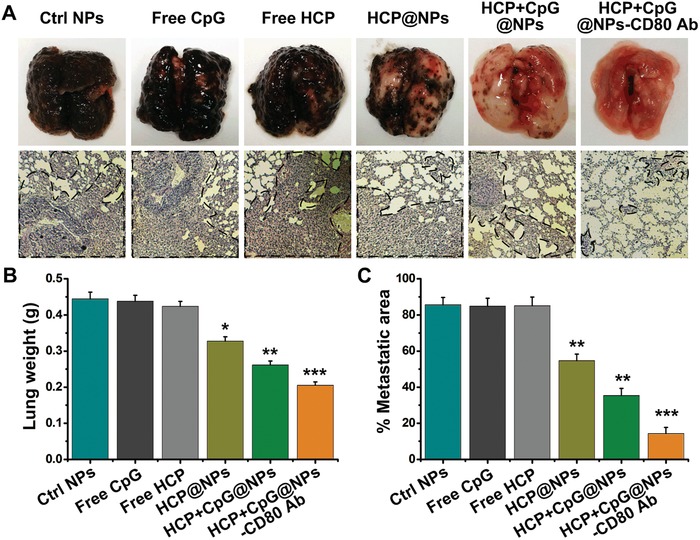
In vivo antimetastatic effect in B16F10 tumor‐bearing mice. A) Representative images (upper panel) and H&E‐stained sections (magnification 200 ×) (bottom panel) of lung tissues after intravenous injections of different modified NPs (*n* = 4). Black spots (upper panel) and dashed outlines (bottom panel) indicate lung metastases. B) Quantitative analysis of the entire weight of lungs from (A). C) Quantitative analysis of the metastatic area of lung sections from (A). *P < 0.05, **P < 0.01, and ***P < 0.001.

To evaluate the effect of the formed EAC‐NPs on tumor metastasis in vivo, mice were inoculated subcutaneously with B16F10 tumor cells on day 0, and the different modified NPs were injected intravenously on days 4, 6, and 8. The degree of metastasis was significantly reduced in EAC‐NP‐treated mice (**Figure** **8**A upper panel). This was also demonstrated by H&E staining (Figure 8A bottom panel). Additionally, the entire weight of the lung tissue (Figure 8B) and the area of metastasis (Figure 8C) also decreased significantly. These results confirm that the formed EAC‐NPs could greatly inhibit the progression of tumor metastasis.

## 3 Conclusion

A major challenge of producing antitumor vaccines is the inefficient uptake of antigens and adjuvants by APCs in peripheral tissue. As a result, the processing and presentation of tumor antigens to T cells and the subsequent induction of specific antitumor immune responses are hampered.^36^ Here, we developed biodegradable and biocompatible EAC‐NPs that are easy to produce through self‐assembly of hydrophilic and lipophilic constitutive di‐block copolymers. We showed that EAC‐NPs targeted blood APCs, and a small amount of EAC‐NPs (500 µg, containing 9.03 µg HCP, 1.25 µg CpG, and 0.4 µg CD80 Ab) enhanced the efficient uptake and presentation of HCP by APCs. This resulted in a more robust activation of CD8^+^ and CD4^+^ T cells and tumor regression without causing adverse events in vivo. The combined tumor antigen HCP and immune adjuvant CpG complemented each other in the activation of APCs, and resulted in a synergistic biological effect that altered the course of T cell responses and inhibited tumor growth and metastasis.

All in all, four advantages exist in this system: 1) EAC‐NPs can generate potent T cell immune responses against a variety of tumors (e.g., mastadenoma and melanoma), which depend on the source of endogenous tumor antigens (HCP). Thus, the functionalized EAC‐NPs for immunotherapy are more effective for tumors with low immunogenicity. In cancer patients, antigenic HCP can be obtained from excised tumors or extracted ascites. 2) In vitro loading of HCP, which is a mixture of multiple endogenous antigens, with a low concentration in combination with CpG, induces a synergistic T cell‐mediated antitumor immune response. Consequently, immune tolerance caused by high antigen concentration and inadequate immune response caused by low antigen concentration can be avoided. 3) The immunotherapy has not only been used for treating cancers but has also been used to treat other antigen‐related diseases, such as infections and inflammation. 4) EAC‐NPs are easy to produce, and virtually any tumor antigen can be obtained from HSP70‐peptide complexes in patient tumor tissues. Therefore, our work can provide an ideal platform for precision medicine with personalized immunotherapy and practical way to treat cancer.

## 4 Experimental Section

*Isolation and Analysis of HCP*: Tumor tissues were dissociated by mechanical separation and used to generate single cell suspensions using a 74 µm cell strainer. The cells were then resuspended in ACK lysis buffer to remove red blood cells. Tumor cells were homogenized in 40 mL of lysis buffer (50 × 10^−3^ m Tris‐HCl, pH 8.0, 1% Triton X‐100, 150 × 10^−3^ m NaCl, 100 µg mL^−1^ phenylmethylsulfonyl fluoride) supplemented with protease inhibitor cocktail set I for 50 min on ice. After centrifugation (4 °C, 12 000 rpm, 20 min), the solubilized proteins were immunoprecipitated with anti‐HSP70 antibody followed by protein A/G‐Sepharose beads (Roche) at 4 °C overnight. Then, the final pellet of protein/antibody/bead complexes was extensively washed with lysis buffer. The complexes were incubated with 10 × 10^−3^ m ATP at room temperature for 30 min. The supernatant was collected, and the protein concentration was quantified using the Bradford assay. The protein was then mixed with 5 × loading dye and separated by SDS‐PAGE. Finally, the gel was stained with Coomassie blue G250.

*Formulation of HCP and CpG‐Loaded PCL‐Hyd‐PEG Vesicles*: 5 mg PCL_5k_‐Hyd‐PEG_4k_‐COOH copolymer (Figure S8, Supporting Information) was dissolved in 500 µL THF. The mixture of HCP (100 µg 50 µL^−1^) and CpG ODNs (2.0 nmol 50 µL^−1^) was adjusted by adding dilute hydrochloric acid until the pH was 7.5. This mixture was then added to the copolymer solution at a rate of 1 drop 15 s^−1^ with vigorous stirring. After 2 h of stirring at room temperature, the formed solution was mixed with 1‐ethyl‐3‐(3‐dimethylaminopropyl)‐carbodiimide (EDC, 100 × 10^−3^ m, 50 µL) and N‐hydroxysuccinimide (NHS, 100 × 10^−3^ m, 100 µL) for 4 h to activate the carboxyl groups. The CD80 antibody (CD80 Ab) (0.1667 nmol 50 µL^−1^) was then added to the solution and stirred for 24 h at room temperature to form the link between the antibody and the vesicles. The formed NP solution was centrifuged at 300 rpm for 15 min, then washed twice by deionized water, and finally dialyzed (MWCO = 8000 Da) to remove small molecules.

*Flow Cytometry*: To evaluate the functional properties of tumor‐infiltrating lymphocytes, the tumor tissues were excised after the animals were euthanized, washed in complete RPMI 1640 medium, and disaggregated using a 74 µm cell strainer. The cells were then washed twice with PBS+1.0% FBS. Red blood cells were then removed using ACK lysis buffer. Tumor‐infiltrating lymphocytes were obtained using the lymphocyte separation liquid (LTS1092Z, TBDsciences, Tanjin, China) and then stained with designated antibodies. The samples were analyzed with a FACS Canto flow cytometer (Becton Dickinson, Mountain View, CA).

*Uptake and Distribution of NPs by Macrophages and DCs*: RAW264.7 and DC2.4 cells were seeded in confocal Petri dishes and grown for 12 h at 37 °C in 5% CO_2_ and then incubated with HCP+CpG@NPs or HCP+CpG@NPs‐CD80 Ab (HCP labeled by Cy5‐NH_3_) for 2 h. Cells were washed three times with PBS and stained with Lyso‐Tracker Red and ER‐Tracker Green to indicate the Lyso and ER, respectively. The uptake and colocalization of HCP were observed using confocal laser scanning microscopy (LSM 510 META, Carl Zeiss, Jenna, Germany).

*Cytotoxicity of NPs*: Blood monocytes and BMDCs were seeded in 96‐well plates (2.5 × 10^4^ cells per well) in 100 µL RPMI 1640 media containing 10% FBS, 100 U mL^−1^ penicillin, and 100 µg mL^−1^ streptomycin for 12 h and then incubated with different concentrations of HCP+CpG@NPs‐CD80 Ab (0, 100, 250, and 400 µg mL^−1^). After 24 h, the medium was replaced with 100 µL fresh serum‐free medium containing 10 µL Cell Counting Kit‐8 (CCK‐8) reagent (Dojindo). Finally, cell viability was detected after incubation for 4 h at 37 °C using a microplate reader (Infinite 200, TECAN, Switzerland). Cell viability (%) was calculated using the following equation: cell viability (%) = (A_treated_/A_control_) × 100%, where A_treated_ and A_control_ represented the absorbance values (at 450 nm) of HCP+CpG@NPs‐CD80 Ab‐treated cells and untreated cells, respectively.

*Ability of NPs to Stimulate the Production of IFN‐γ In Vivo*: To determine the ability of various formulations to stimulate the production of IFN‐γ by CD8^+^ and CD4^+^ T cells in splenocytes, BALB/c mice aged 6–8 weeks were administered intravenously with PCL‐Hyd‐PEG NPs, Free CpG, Free HCP, HCP@PCL‐Hyd‐PEG NPs, HCP+CpG@PCL‐Hyd‐PEG NPs, or HCP+CpG@PCL‐Hyd‐PEG‐CD80 Ab NPs (500 µg NPs 100 µL^−1^) on days 4, 6, and 8 after tumor implantation. Splenocyte suspensions were prepared from the mice on day 16, and the cells were purified using lymphocyte separation liquid. Then, the spleen lymphocytes were fixed, permeabilized and incubated with anti‐IFN‐γ antibody and measured using flow cytometry.

*Tumor Therapy In Vivo*: To evaluate the therapeutic efficacy of HCP+CpG@NPs‐CD80 Ab in vivo, mice bearing EMT6 or 4T1 tumors were randomized into six groups: PCL‐Hyd‐PEG NP group, Free CpG group, Free HCP group, HCP@PCL‐Hyd‐PEG NP group, HCP+CpG@PCL‐Hyd‐PEG NP group, and HCP+CpG@PCL‐Hyd‐PEG‐CD80 Ab NP group. Treatments were administered intravenously to tumor‐bearing mice on days 4, 6, and 8. Tumor dimensions were measured every 2 d using digital calipers beginning on day 5. Mouse survival and weight were monitored daily, and tumor volume was calculated using the formula: volume (mm^3^) = 0.5 × length × width^2^, where length is the largest diameter and width is the perpendicular diameter. The mice were euthanized when the tumor volume exceeded 2.5 cm^3^ or 10% of their body weight. The tumors and main organs of the mice were harvested for flow cytometry analysis after various treatments. The study was approved by the Institutional Animal Care and Use Committee of the university (South China Normal University, Guangzhou, China).

*Tumor Metastasis Analysis*: The antimetastasis activity of the HCP+CpG@NPs‐CD80 Ab was evaluated by metastatic nodules. In brief, the mice with established B16F10 lung metastases by subcutaneous and intravenous injection^37^ were treated intravenously with PCL‐Hyd‐PEG NPs, Free CpG, Free HCP, HCP@PCL‐Hyd‐PEG NPs, HCP+CpG@PCL‐Hyd‐PEG NPs, or HCP+CpG@PCL‐Hyd‐PEG‐CD80 Ab NPs on days 4, 6, and 8. After 32 d, the mice were sacrificed and the tumor‐loaded lungs were excised. The lungs were weighed and then stained by H&E to detect the formation of micrometastatic foci. The metastatic area of every tissue section was analyzed using Image‐Pro Plus 6.0 software (Media Cybernetics, Bethesda, MD).

*Statistical Analysis*: All quantitative data were expressed as mean ± SEM from triplicate measurements and were analyzed statistically using a One‐way ANOVA to compare more than two groups or Student's *t*‐test (two‐tailed) to compare two groups of independent samples, unless otherwise noted. A value of *P* < 0.05 was considered statistically significant.

## Conflict of Interest

The authors declare no conflict of interest.

## Supporting information

Supporting Information
