# Supplementary material for: Targeting and Specific Activation of Antigen‐Presenting Cells by Endogenous Antigen‐Loaded Nanoparticles Elicits Tumor‐Specific Immunity
Source: Adv Sci (Weinh). 2019 Nov 8;7(1):1900069. doi: 10.1002/advs.201900069 (PMC6947714; doi:10.1002/advs.201900069)
Supplement: Supplementary file 1 — Supporting Information [file ADVS-7-1900069-s001.pdf]

Copyright WILEY-VCH Verlag GmbH & Co. KGaA, 69469 Weinheim, Germany, 2019.

ADVANCED SCIENCE
Open Access

Supporting Information

for *Adv. Sci.,* DOI: 10.1002/advs.201900069

Targeting and Specific Activation of Antigen-Presenting Cells by Endogenous Antigen-Loaded Nanoparticles Elicits Tumor-Specific Immunity

*Hao-Cai Chang, Zheng-Zhi Zou, Qiu-Hong Wang, Jie Li, Huan Jin, Qian-Xia Yin, and Da Xing**

Supporting Information

Targeting and Specific Activation of Antigen-Presenting Cells by Endogenous Antigen-Loaded Nanoparticles Elicits Tumor-Specific Immunity

Hao-cai Chang, Zheng-zhi Zou, Qiu-hong Wang, Jie Li, Huan Jin, Qian-xia Yin, Da Xing*

Dr. H.-C. Chang, Dr. Z.-Z. Zou, Q.-H. Wang, Dr. J. Li, H. Jin, and Q.-X. Yin, Prof. D. Xing

MOE Key Laboratory of Laser Life Science & Institute of Laser Life Science

South China Normal University

Guangzhou 510631, China

E-mail: xingda@scnu.edu.cn

Dr. H.-C. Chang, Dr. Z.-Z. Zou, Q.-H. Wang, Dr. J. Li, H. Jin, and Q.-X. Yin, Prof. D. Xing

College of Biophotonics

South China Normal University

Guangzhou 510631, China

1. Experimental Section

Materials: PCL_{5k}-Hyd-PEG_{4k}-COOH was purchased from Xi'an Ruixi Biological Technology Co. Ltd. (Xi'an, China). CpG ODN 1826 (5'-tccatgacgttcctgacgtt-3') (tlrl-1826-5) and FITC-labeled CpG ODN (5'-tccatgacgttcctgacgtt-3') (tlrl-1826f) were purchased from InvivoGen (San Diego, CA). Cy7-labeled CpG ODN (5'-tccatgacgttcctgacgtt-3') was synthesized by Sangon Biotech (Shanghai, China). Lyso-Tracker Red (C1046) was purchased from Beyotime Biotechnology (Jiangsu, China). ER-Tracker Green (BODIPY® FL Glibenclamide) (E34251), Pierce™ Protein A/G Agarose (20421) and ATP (PV3227) were purchased from Thermo Fisher Scientific (Waltham, MA). Cyanine 5 amine (ab146463) and Cyanine 7 amine (ab146465) were purchased from Abcam (Cambridge, MA). IL-12p70 (KA0244) and TNFα (KA0257) ELISA kit were purchased from Abnova Corporation (Taipei, Taiwan).

The following antibodies were used: anti-HSP70 (Santa cruz, sc-24), anti-CD80 APC (Biolegend, 104714), anti-CD80 FITC (Biolegend, 104705), anti-CD3 APC (Biolegend, 100236), anti-CD8 PE (Biolegend, 100707), anti-CD4 PE/Cy7 (Biolegend, 100421), anti-CD25 PE (Biolegend, 101904), anti-FOXP3 Alexa Fluor 488 (Biolegend, 320012), anti-CD11b PE/Cy7 (Biolegend, 101216), anti-F4/80 PE/Cy7 (Biolegend, 123113), anti-CD11c PE/Cy7 (Biolegend, 117318), anti-CD44

APC (Biolegend, 103011), anti-CD122 (IL-2Rβ) FITC (Biolegend, 123207), anti-IL-12 p40 PE (Biolegend, 505203), anti-MHC I FITC (Biolegend, 114606), anti-MHC II PE (Biolegend, 109908), anti-TNF α FITC (Miltenyi Biotec GmbH, 130-109-765), anti-IFN-γ PE (Miltenyi Biotec GmbH, 130-109-769), anti-IFN-γ FITC (Miltenyi Biotec GmbH, 130-109-768) and anti-CD69 FITC (Miltenyi Biotec GmbH, 130-103-983).

Cell culture: 5- to 7-week-old BALB/c mice were injected intraperitoneally (i.p.) with 1 mL of 3% thioglycolate. After 2 d, the peritoneal cells were collected by peritoneal lavage with 10 mL of cold serum-free RPMI 1640 medium (Invitrogen, Carlsbad, CA). The cells were cultured for 5 h and the peritoneal macrophages (Mφ) adhered to plates, all nonadherent cells were subsequently removed by washing with PBS, and then the adherent cells were used for our experiments.

(1×10^7 cells per well) were seeded into 6-well plates and incubated in 4 mL RPMI 1640 supplemented with heat-inactivated FBS (10%, Invitrogen), IL-4 (50 ng mL^{-1}, Peprotech) and the growth factors GM-CSF (50 ng mL^{-1}, Peprotech). After incubation for 24 h, nonadherent cells were removed with fresh medium, while half of the medium was replaced every two days and new medium supplemented with IL-4 and GM-CSF. On day 7, nonadherent cells and loosely adherent cells (bone marrow-derived dendritic cells, BMDCs) in the culture supernatant were harvested and used for subsequent experiments.

Splenic T lymphocytes were harvested from spleens and then resuspended in ACK lysis buffer to remove red blood cells. T lymphocytes were purified using lymphocyte separation liquid (TBDsciences, LTS1092PK). All primary cells were cultured in RPMI 1640 supplemented with 10% heat-inactivated FBS, 100 μg mL^{-1} streptomycin (Invitrogen) and 100 U mL^{-1} penicillin (Invitrogen) in a humidified incubator at 37°C containing 5% CO_2.

Blood monocytes were isolated immediately using mice peripheral blood monocytes isolation kit (TBD2011M, TBDsciences, China) according to its instructions. And then the cells were cultured in complete RPMI-1640 supplemented with 10% heat-inactivated FBS, 100 μg mL^{-1} streptomycin and 100 U mL^{-1} penicillin in a humidified incubator at 37°C containing 5% CO_2.

Murine cell lines 4T1, EMT6 and B16F10 were maintained in Dulbecco's Modified Eagle Medium (DMEM, Invitrogen, Carlsbad, CA) supplemented with 10% FBS, 100 μg mL^{-1} streptomycin, and 100 U mL^{-1} penicillin. RAW264.7 and DC2.4 cell lines were maintained in RPMI 1640 medium supplemented with 10% FBS, 100 μg mL^{-1} streptomycin, and 100 U mL^{-1} penicillin.

Size, zeta potential, and morphology of vesicles: The size and zeta potential of the vesicles were measured on a ZetaSizer Nano ZS90 (Malvern Instrument, Worcs, UK). The morphology of the vesicles was observed by transmission electron microscopy (TEM, JEOL JEM-2100HR). Before observation, drops of the

suspensions were deposited onto a copper grid coated with carbon film and dried at room temperature.

Quantitative measurement of the HCP, CpG, and CD80 Ab on vesicles: To quantitatively measure the HCP in vesicles, the suspension of the HCP+CpG@NPs-CD80 Ab vesicles was centrifuged and washed with deionized water three times. The vesicles were then broken by ultrasonic treatment and the protein concentration was measured using the Bradford assay. The obtained standard curve of BSA was y = 0.38277x + 0.00059 (y: absorbance value at 595 nm; x: concentration of BSA; R^2 = 0.99553). According to the absorption value of the samples at 595 nm, the calculated concentration of HCP was 18.06 ± 0.84 μg per 1 mg PCL-Hyd-PEG vesicles. HCP loading efficiency was calculated according to the following formula: HCP loading efficiency (%) = (weight of encapsulated HCP/weight of initial HCP added) × 100%.

To measure the amount of CpG and CD80 Ab on vesicles, CpG-FITC and CD80 Ab-APC were used to replace CpG and CD80 Ab. The absorption spectra of CpG-FITC and CD80 Ab-APC samples with different concentrations were obtained using a UV-vis spectrometer. The obtained standard curve of CpG-FITC was y = 0.1212x (y: absorbance value at 488 nm; x: concentration of CpG-FITC; R^2 = 0.9743) (Figure S9A, Supporting Information). According to the absorption value of CpG-FITC at 488 nm and with HCP@NPs-CD80 Ab as a blank control,

the calculated concentration of CpG was 0.366 ± 0.012 nmol per 1 mg PCL-Hyd-PEG vesicles. The obtained standard curve of CD80 Ab-APC was y = 0.0379x (y: absorbance value at 650 nm; x: concentration of CD80 Ab-APC; R^2 = 0.9083) (Figure S9B, Supporting Information). According to the absorption value of CD80 Ab-APC at 650 nm and with HCP+CpG@NPs as a blank control, the calculated concentration of CD80 Ab was 13.3 ± 0.21 pmol per 1 mg PCL-Hyd-PEG vesicles.

***In Vitro* stability of HCP+CpG@NPs-CD80 Ab vesicles in PBS, DMEM, FBS and DMEM+FBS:** HCP+CpG@NPs-CD80 Ab vesicles incubated in PBS (0.01 M, pH 7.4), DMEM, FBS and DMEM with FBS (10%) at 4°C for different times and the change of nanoparticle size was assessed by DLS.

Evaluation of pH-sensitive vesicles: To confirm that the hydrazone bonds of these vesicles were cleavable under mildly acidic conditions, the vesicles were incubated with PBS at various pHs (0.01 M PBS at pH 5.0, 6.0, and 7.4) over 10 h at room temperature. The change in nanoparticle size was then assessed by DLS.

2. Supporting Figures

Table S1. Mean zeta potentials and mean hydrodynamic diameter (D_h) of PCL-Hyd-PEG NPs, HCP@PCL-Hyd-PEG NPs, HCP+CpG@PCL-Hyd-PEG NPs, and HCP+CpG@PCL-Hyd-PEG-CD80 Ab NPs determined by DLS techniques.

NPs	Zeta potential [mV]	PDI	D_h [nm]
PCL-Hyd-PEG	-9.0 ± 2.1	0.125	139 ± 2
HCP@PCL-Hyd-PEG	-9.2 ± 3.2	0.159	142 ± 5
HCP+CpG@PCL-Hyd-PEG	-10 ± 2.5	0.205	143 ± 6
HCP+CpG@PCL-Hyd-PEG-CD80 Ab	-15 ± 3.3	0.215	150 ± 5

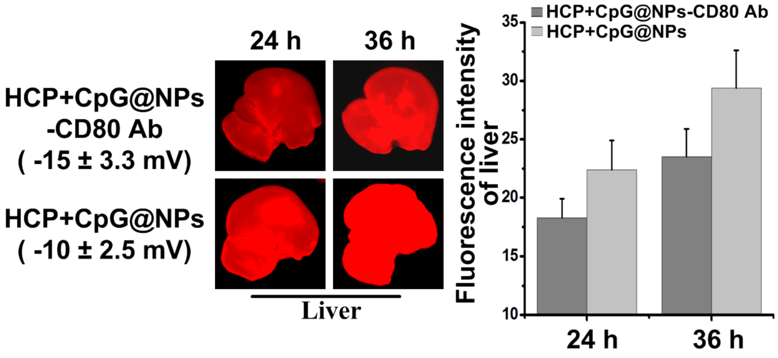

Figure S1. *Ex vivo* imaging of livers from mice 24 h and 36 h after intravenous injection of HCP+CpG@NPs-CD80 Ab and HCP+CpG@NPs (HCP labeled by Cy7-NH_3) was visualized and the fluorescent images of livers were quantitatively analyzed using an Odyssey infrared imaging system.

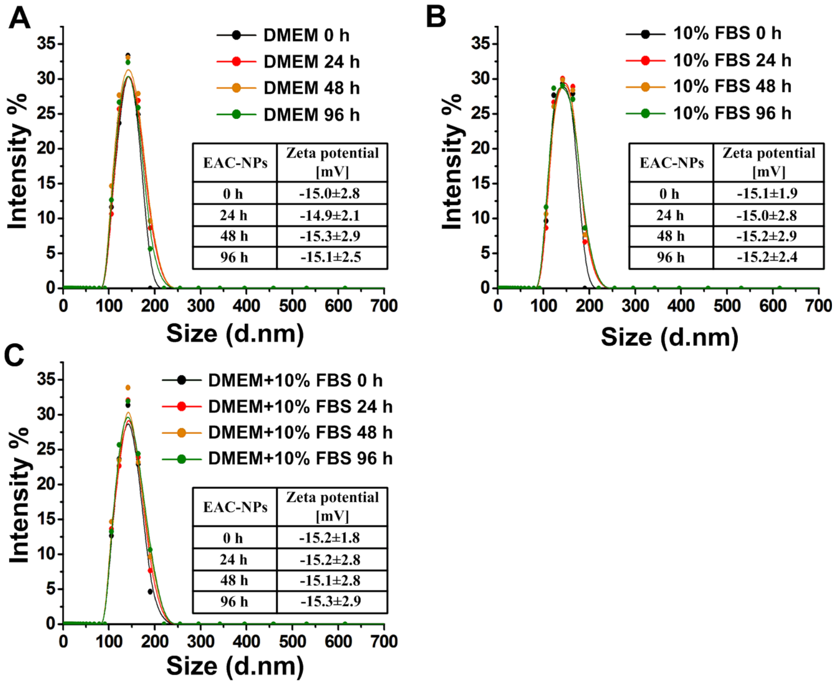

Figure S2. Size distribution and zeta potentials of HCP+CpG@NPs-CD80 Ab vesicles in DMEM **(A)**, FBS **(B)** and DMEM with FBS (10%) **(C)** at different time points.

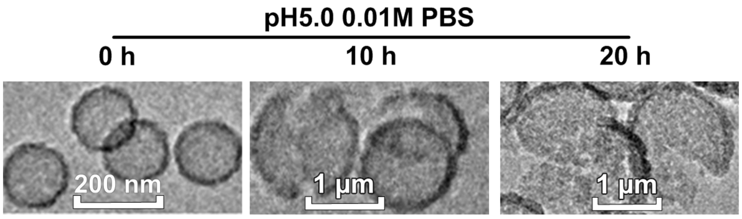

Figure S3. Transmission electron microscopy images of EAC-NPs in 0.01 M PBS at pH 5.0 after 0 h, 10 h and 20 h, respectively.

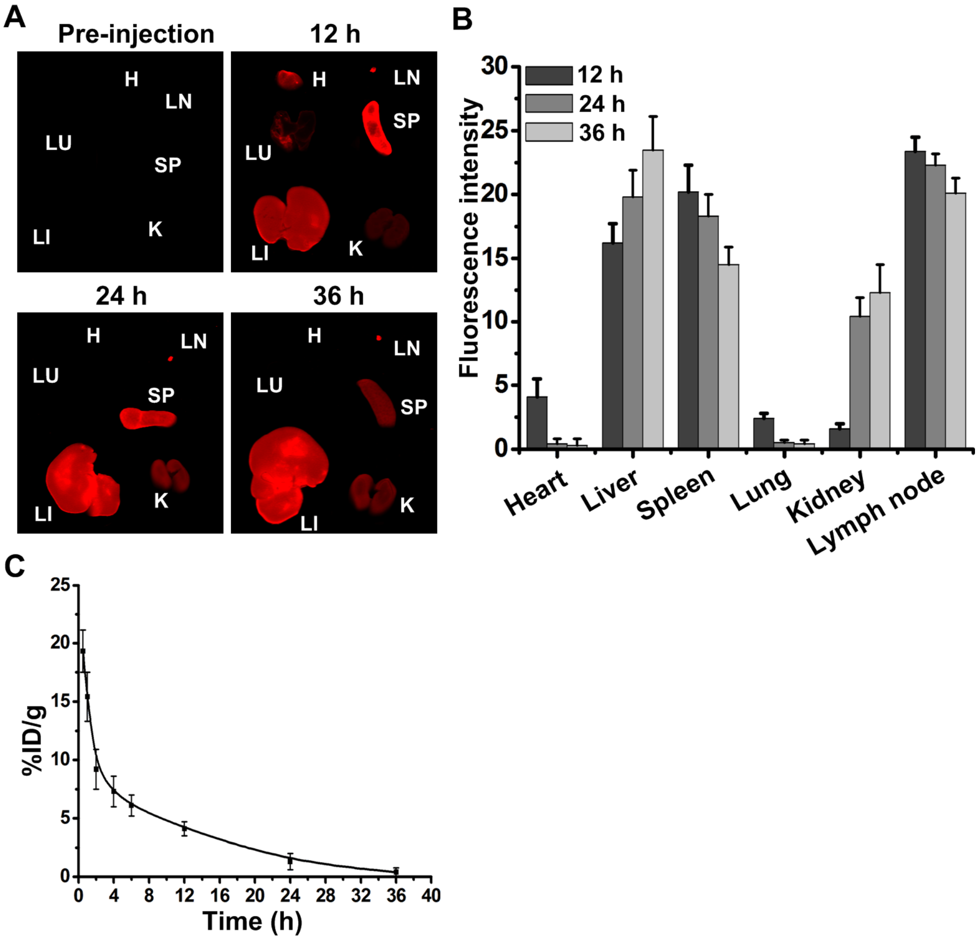

Figure S4. (A) Spectrally resolved *ex vivo* fluorescence images of organs before injection and 12 h, 24 h, and 36 h after injection of EAC-NPs (HCP labeled by Cy7-NH_3). H: heart, LI: liver, SP: spleen, LU: lung, K: kidney, LN: lymph nodes. **(B)** Quantitative biodistribution of EAC-NPs in each organ was analyzed using an Odyssey infrared imaging system. **(C)** The blood circulation curve of EAC-NPs determined by measuring APC fluorescence (CD80 Ab-APC) in the blood at different time points post injection in healthy mice (n = 4). % ID/g was a percentage of the injected dose per gram of tissue.

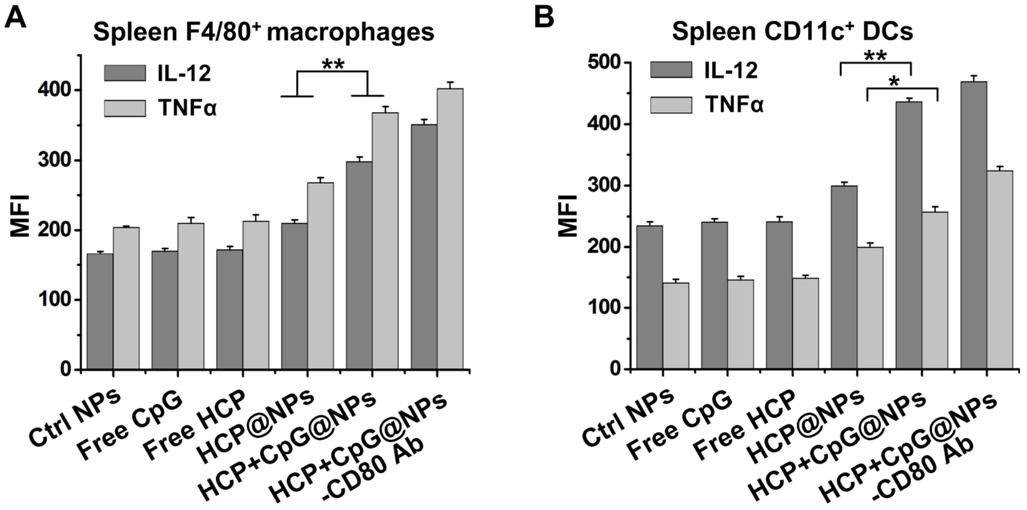

Figure S5. (**A-B**) Flow cytometry analysis evaluated the IL-12 and TNF α expression in F4/80$^+$ macrophages (**A**) and CD11c$^+$ DCs (**B**) in spleens of animals under different treatments (n = 4). *P < 0.05 and **P < 0.01.

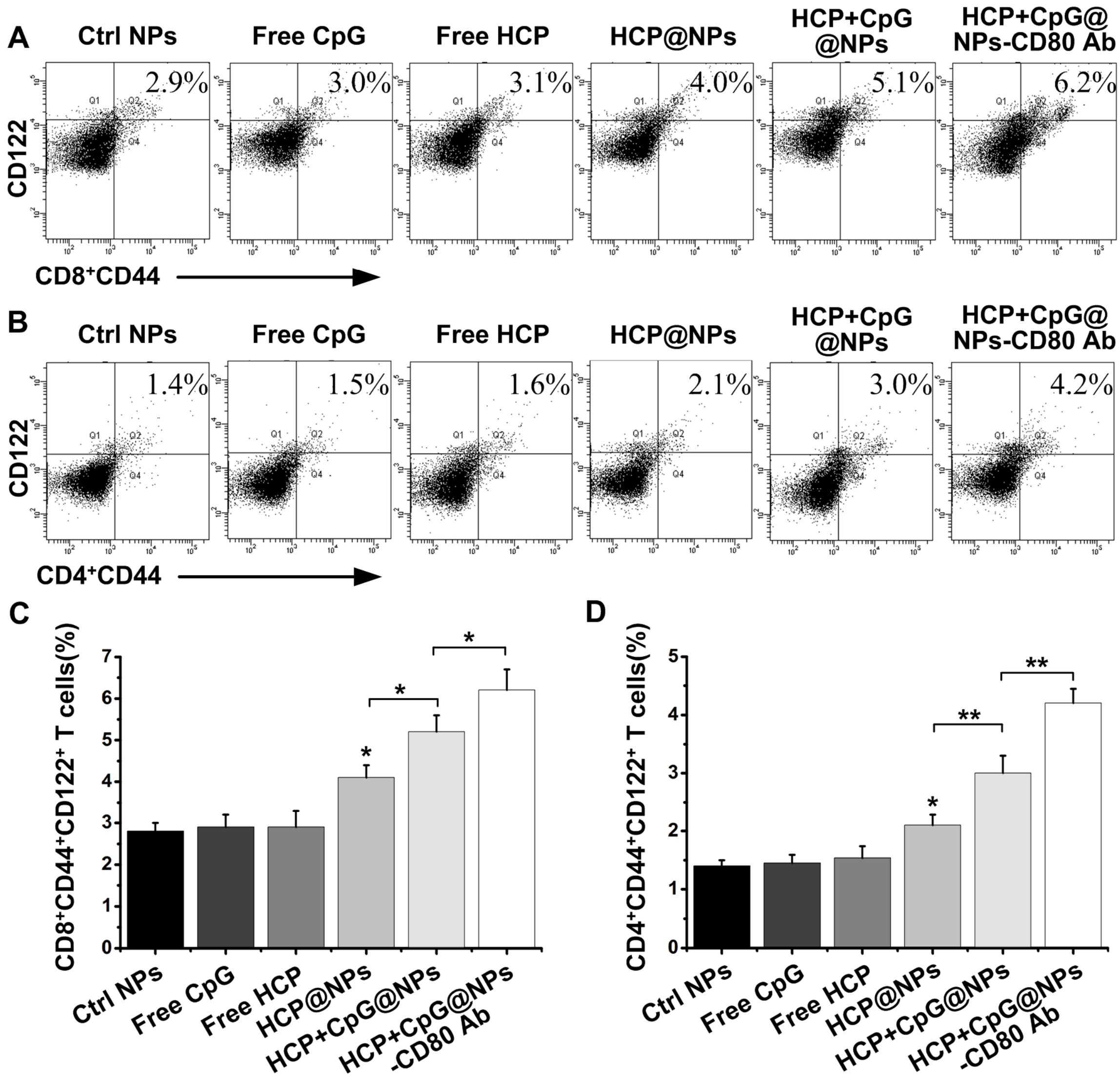

Figure S6. Flow cytometry analysis was used to assess the abundance of $CD8^+CD44^+CD122^+$ (A) and $CD4^+CD44^+CD122^+$ (B) memory T cells in spleen of 4T1 tumor-bearing mice treated with different modified NPs 18 d after tumor inoculation (n = 4). (C-D) Quantitative statistical results of $CD8^+CD44^+CD122^+$ (C) and $CD4^+CD44^+CD122^+$ (D) memory T cells from (A) and (B). $*P < 0.05$ and $**P < 0.01$.

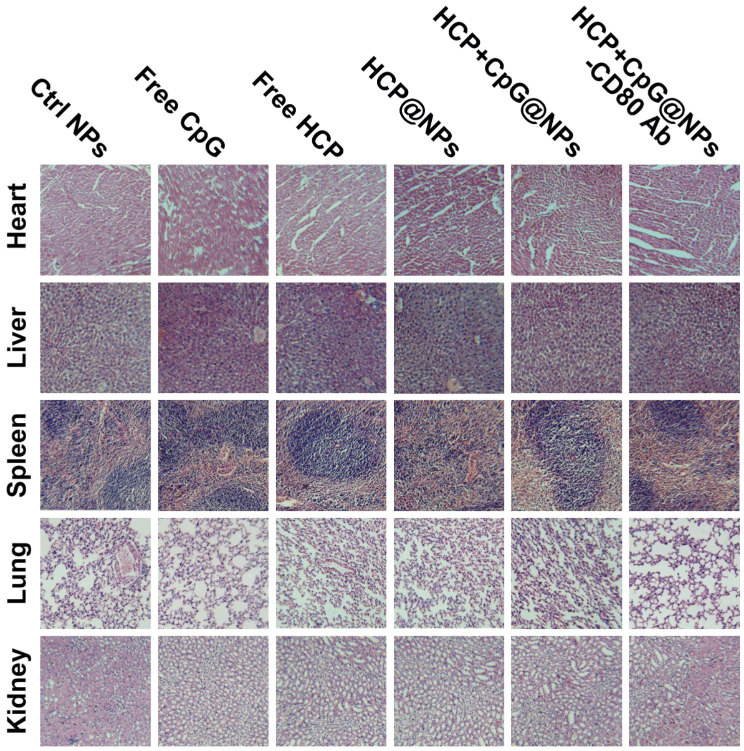

Figure S7. The H&E stained images of main organs collected from healthy mice treated with different modified NPs (magnification 200×).

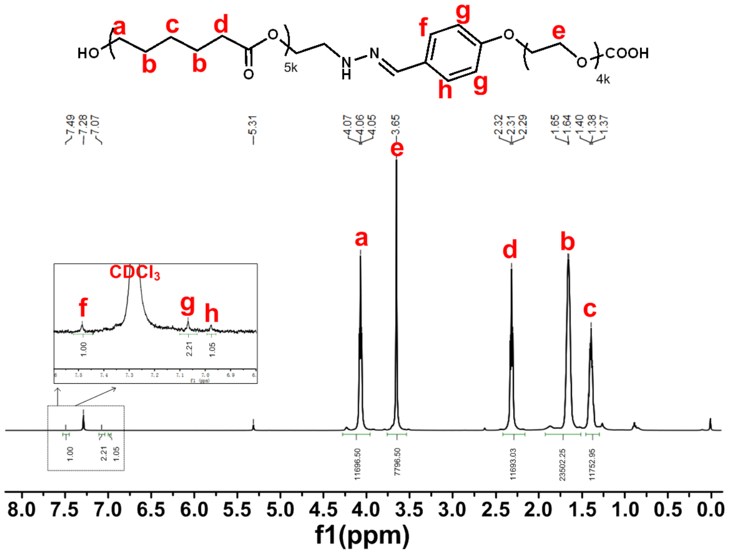

Figure S8. ^{1}H NMR spectrum of PCL_{5k}-Hyd-PEG_{4k}-COOH in $CDCl_3$.

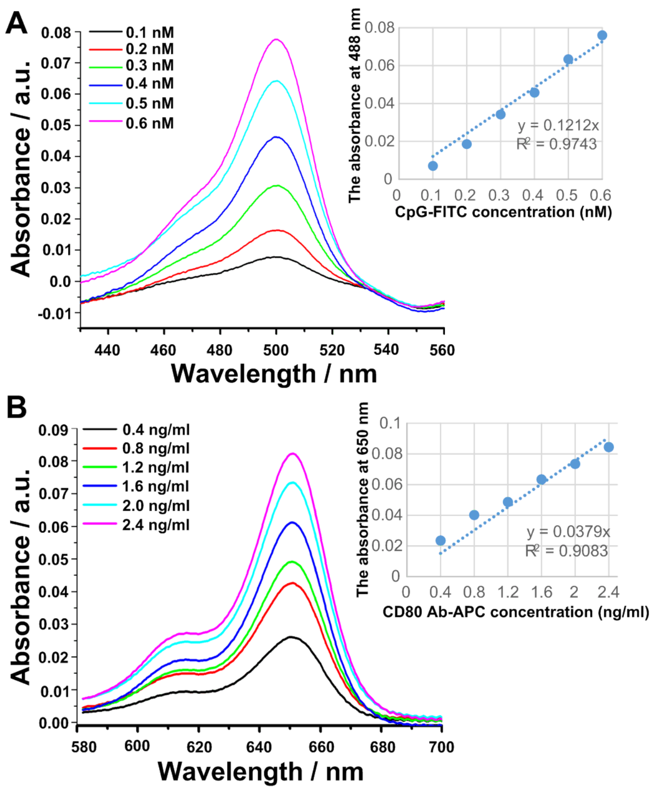

Figure S9. The quantification curves of CpG and CD80 Ab. **(A)** The UV-VIS-NIR spectra of CpG-FITC (488 nm) with different concentrations. **(B)** The UV-VIS-NIR spectra of CD80 Ab-APC (650 nm) with different concentrations.
